# Mechanical Thrombectomy for TRACE‐III‐Eligible Patients With Ischemic Stroke: A Multicenter Retrospective Study Compared to TRACE‐III and TIMELESS Trials

**DOI:** 10.1002/acn3.70107

**Published:** 2025-08-16

**Authors:** Chongyang Huang, Yanru Liu, Jiangang Zhang, Sheng Guan, Tao Quan, Zhen Chen, Xiaojie Fu, Sen Wei, Kaihao Han, Xiaoan Zhou, Chengcheng Zhu, Edgar Samaniego, Yueqi Zhu, Haowen Xu

**Affiliations:** ^1^ Department of Neuro‐Interventionalradiology The First Affiliated Hospital of Zhengzhou University Zhengzhou China; ^2^ Neurology The First Affiliated Hospital of Zhengzhou University Zhengzhou China; ^3^ Department of Neurology Anyang Central Hospital Anyang China; ^4^ College of Health and Human Science, Purdue University West Lafayette Indiana USA; ^5^ Department of Radiology University of Washington Seattle Washington USA; ^6^ Yale School of Medicine and Yale University New Haven Connecticut USA; ^7^ Department of Radiology Shanghai Sixth People's Hospital Affiliated to Shanghai Jiao Tong University School of Medicine Shanghai China

**Keywords:** intravenous thrombolysis, ischemic stroke, mechaniclal thrombectomy, perfusion imaging

## Abstract

**Objective:**

The results of the TRACE‐III trial demonstrated that tenecteplase (TNK) might be comparable to TNK combined with mechanical thrombectomy (MT) for large vessel occlusion (LVO) stroke within 4.5 to 24 h of onset, as tested in the TIMELESS trial. We aimed to evaluate the safety and effectiveness of MT alone in TRACE‐III‐eligible patients in routine clinical settings, comparing the results of both trials.

**Methods:**

This retrospective multicenter cohort study involved consecutive patients who underwent MT alone at three stroke centers between March 2021 and July 2024. Only those meeting the inclusion and exclusion criteria of TRACE‐III were included. Safety, clinical, and imaging outcomes were compared with the TRACE‐III and TIMELESS trial data.

**Results:**

336 TRACE‐III‐eligible patients were enrolled in our cohort. MT alone had a higher percentage of patients with a modified Rankin scale (mRS) score of 0–2 at 90 days (58.9%) compared to the TNK group in TRACE‐III, TNK and placebo groups in TIMELESS (43.6%, 46.0%, and 42.4%, respectively; all *p* < 0.01). Additionally, MT achieved a higher rate of early‐stage reperfusion (86.0%) compared to the TRACE‐III TNK, TIMELESS TNK, and TIMELESS placebo groups (27.9%, 76.7%, and 63.9%; all *p* < 0.05). The mRS 0–1 at 90 days, incidence of symptomatic intracerebral hemorrhage, and mortality at 90 days were 36.0%, 5.4%, and 14.9%, respectively, similar to those in the three groups (all *p* > 0.05).

**Interpretation:**

In clinical practice, MT can achieve higher rate of early‐stage reperfusion and tended to yield better functional outcomes than intravenous TNK, with similar safety, in TRACE‐III‐eligible patients.

## 1 Introduction

Acute ischemic stroke (AIS) is a leading cause of mortality and disability in adults. Timely recanalization of the occluded artery is an effective treatment of AIS [1]. Over the past decade, significant progress has been made in reperfusion treatment. The efficacy and safety of intravenous thrombolysis with alteplase or tenecteplase (TNK) for large vessel occlusion (LVO) stroke within 4.5 h of onset have been established [2, 3, 4]. Mechanical thrombectomy (MT) has emerged as a first‐line treatment for LVO stroke up to 24 h from onset [5, 6, 7]. In the past few years, three studies have extended alteplase thrombolysis up to 9 h and for wake‐up stroke using perfusion imaging selection, achieving better functional outcomes compared to placebo [8, 9, 10].

In 2024, two trials extended the treatment time window of TNK to 24 h by using perfusion imaging for patient selection. The Tenecteplase Reperfusion Therapy in Acute Ischemic Cerebrovascular Events‐III (TRACE‐III) trial randomized patients with anterior circulation LVO and salvageable brain tissue identified on perfusion imaging, who did not have access to thrombectomy, to receive TNK or standard medical treatment [11]. The TRACE‐III trial demonstrated an absolute benefit of nearly 10% in achieving a modified Rankin Scale (mRS) score of 0–1 for patients treated with TNK. In the TNK arm, 33.0% achieved an excellent outcome (mRS 0–1 at 90 days), and 43.6% had a good outcome (mRS 0–2 at 90 days). The Thrombolysis in Imaging Eligible, Late Window Patients to Assess the Efficacy and Safety of Tenecteplase (TIMELESS) trial showed that TNK plus MT between 4.5 and 24 h after stroke onset in patients with middle cerebral artery or internal carotid artery occlusions and perfusion mismatch did not appear to be more effective or safer than placebo plus MT [12]. In the TNK group, 32.3% of patients achieved excellent outcomes, and 46.0% had good outcomes, compared to 26.6% and 42.4% in the placebo group, respectively. Safety outcomes including sICH and mortality were similar to those in the TRACE‐III TNK arm.

The inclusion criteria for the two trials were similar, prompting a question of whether intravenous TNK could serve as an alternative to MT for patients selected by perfusion imaging within the 4.5‐ to 24‐h window. However, the TIMELESS trial did not fully address this question, since approximately 23% of patients did not undergo MT after receiving TNK or placebo. To investigate further, we conducted a multicenter retrospective study in a real‐world setting, focusing on patients who met the TRACE‐III inclusion and exclusion criteria. Our aim was to compare the safety and efficacy of MT alone in our cohort with TNK in TRACE‐III and TNK or placebo in TIMELESS. The results will determine the necessity for additional randomized trials.

## 2 Methods

### 2.1 Patients and Study Design

This study was conducted in accordance with the Ethical Guidelines for Medical and Health Research Involving Human Subjects in China and complied with the Declaration of Helsinki guidelines. The institutional review boards from all three hospitals approved this study. Due to its retrospective design, informed consent was waived. The individual deidentified participant data will be shared on request to the corresponding author.

We retrospectively assessed all patients who underwent CT/MR perfusion imaging followed by MT for AIS caused by anterior circulation LVO from March 2021 to July 2024 at three comprehensive stroke centers in China: The First Affiliated Hospital of Zhengzhou University, Shanghai Sixth People's Hospital Affiliated to Shanghai Jiao Tong University School of Medicine and Anyang People's Hospital. LVO was identified via baseline computed tomography angiography (CTA) or magnetic resonance angiography (MRA) as occlusions in the intracranial internal carotid artery or the first (M1) or second (M2) segments of the middle cerebral artery. Inclusion and exclusion criteria matched those of the TRACE‐III trial, with a comparative analysis presented in Table 1.

**TABLE 1:** Inclusion and exclusion criteria of the present study, TRACE‐III and TIMELESS trial.

Present study	TRACE‐III	TIMELESS
Inclusion criteria		
The same to TRACE‐III trail	Age ≥ 18 years old	Age ≥ 18 years old
The same to TRACE‐III trail	Pre‐stroke mRS score ≤ 1	Pre‐stroke mRS score ≤ 2
The same to TRACE‐III trail	Baseline NIHSS 6–25	Baseline NIHSS 5–42
AIS symptom onset between 4.5 and 24 h prior to enrollment	AIS symptom onset between 4.5 and 24 h prior to enrollment; including wake‐up stroke and unwitnessed stroke, onset time refers to “last‐seen normal time”	AIS symptom onset between 4.5 and 24 h prior to enrollment; including wake‐up stroke and unwitnessed stroke
The same to TRACE‐III trail	Target mismatch profile on CTP or MR + MRP (ischemic core volume < 70 mL, mismatch ratio ≥ 1.8, and mismatch volume ≥ 15 mL)	Target mismatch profile on CTP or MR + MRP (ischemic core volume < 70 mL, mismatch ratio ≥ 1.8, and mismatch volume ≥ 15 mL)
Exclusion criteria		
—	Intention to proceed to endovascular treatment	Active internal bleeding
—	Allergy to tenecteplase	Thrombolytic use in the last 3 months
—	Rapidly improving symptoms at the discretion of the investigator	Hereditary/acquired hemorrhagic diathesis, coagulation factor deficiency
—	Blood glucose < 2.8 or > 22.2 mmol/L	Pre‐existing medical, neurological, or psychiatric disease
The same to TRACE‐III trail	Active internal bleeding or at high risk of bleeding	Severe, uncontrolled hypertension
—	Any known impairment in coagulation due to comorbid disease or anticoagulant use. If on warfarin, then INR > 1.7 or prothrombin time > 15 s	Seizures at stroke onset if it precludes ability to obtain an accurate baseline NIHSS
—	Known defect of platelet function or platelet count below 100,000/mm^3^	Unable to undergo a contrast brain perfusion scan with MR or CT
The same to TRACE‐III trail	Ischemic stroke or myocardial infarction in previous 3 months, previous intracranial hemorrhage, severe traumatic brain injury or intracranial or intraspinal operation in previous 3 months	
The same to TRACE‐III trail	Hypodensity in > 1/3 MCA territory on CT	
The same to TRACE‐III trail	Acute or past ICH identified by CT or MR	
The same to TRACE‐III trail	Multiple arterial occlusion (bilateral MCA occlusion, MCA occlusion accompanied with basilar occlusion).	
Missing clinical or imaging data		
The quality of imaging was poor		

Abbreviations: CT, computerized tomography; CTP, computerized tomography perfusion; ICH, intracerebral hemorrhage; INR, International Normalized Ratio; MCA, middle cerebral artery; MR, magnetic resonance angiography; MRP, magnetic resonance perfusion; mRS, modified Rankin Scale; NIHSS, National Institutes of Health Stroke Scale.

The ischemic core was defined as an area with relative cerebral blood flow less than 30% of normal, measured through CT or MR perfusion imaging. A hypoperfused lesion was determined by a tracer's delayed arrival (time to maximum residue function > 6 s). Evidence of salvageable brain tissue was required and meeting the following criteria: an initial ischemic core volume under 70 mL, an ischemic tissue to infarct volume ratio of at least 1.8, and a penumbra volume of at least 15 mL. Volumes of the ischemic core and penumbra were calculated using the Rapid software (iSchemaView, Menlo Park, California, United States) and the MIStar software (Apollo Medical Imaging, Melbourne, Australia).

### 2.2 Data Acquisition

Baseline demographic data and stroke risk factors, such as hypertension, diabetes mellitus, atrial fibrillation, smoking history, and previous stroke, were documented. Radiological and clinical data were collected at various stages: pre‐procedure, during the procedure, post‐procedure, at discharge, and at a 90‐day follow‐up. Certain parameters, including reperfusion at 24 h post‐MT, major neurological improvement at 72 h, and changes in the NIHSS score at 7 days, were not recorded, as they were absent in most patients' electronic medical records.

### 2.3 Outcomes

To compare the results of our cohort with the TRACE‐III and TIMELESS trials, we aligned our clinical, imaging, and safety outcomes with their primary and secondary focuses. Clinical outcomes were defined as follows: a good functional outcome indicated by a modified Rankin Scale (mRS) score of 0 to 2 at 90 days; an excellent outcome, with an mRS score of 0 to 1 at 90 days; and the ordinal mRS score at 90 days. The mRS was assessed approximately 90 days after MT, with a margin error of ±30 days. Imaging outcomes included successful angiographic recanalization at the end of the procedure, defined by a modified Thrombolysis in Cerebral Infarction (mTICI) score of 2b or 3, and early‐stage reperfusion post‐MT, confirmed by MRA or CTA. Due to the study's retrospective nature, CT/MR angiography for early‐stage reperfusion assessment could be conducted between 24 h and 7 days post‐MT. Safety outcomes included symptomatic intracranial hemorrhage (sICH), defined as intracranial hemorrhage with an increase of ≥ 4 points in the NIHSS score or resulting in death within 36 h post‐MT, and all‐cause mortality at 90 days.

### 2.4 Statistical Analysis

Baseline characteristics were presented as mean and standard deviation (SD) for normally distributed continuous variables, and as median and interquartile ranges (IQRs) for continuous variables. Categorical variables were presented as number and percentage. The *χ* ^2^ test was used to compare our cohort with three groups of TRACE‐III and TIMELESS trials. Due to the unavailability of raw data from these trials, we could not statistically compare continuous variables (e.g., age, baseline NIHSS score) between our cohort and two trials. Missing data were not imputed; instead, a missing category was created for categorical variables. All tests were two‐tailed, with a *p* < 0.05 considered statistically significant. Statistical analysis was performed using a commercial statistical software package (SPSS for Windows, Version 25.0, IBM‐SPSS, Chicago, Illinois, USA).

## 3 Results

### 3.1 Patient Characteristics

**FIGURE 1:**
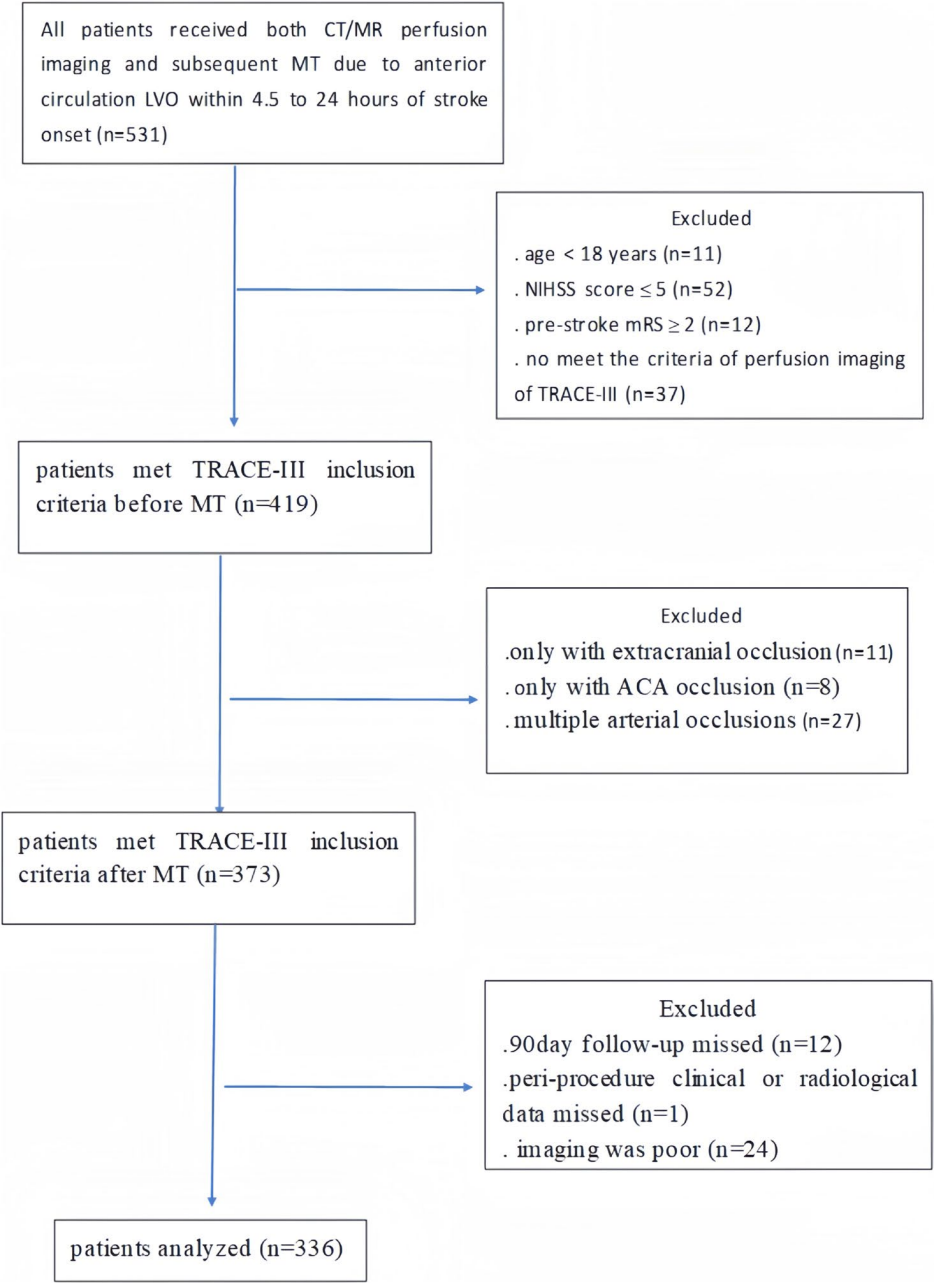
The flow diagram of the patient inclusion process in the study. It outlines the steps involved in enrolling patients for the study. ACA, anterior cerebral artery; CTP, computerized tomography perfusion; ICA, internal carotid artery; MT, mechanical thrombectomy.

Between March 2021 and July 2024, a total of 531 acute ischemic stroke (AIS) patients who underwent both CT/MR perfusion imaging and subsequent MT due to anterior circulation LVO within 4.5 to 24 h of stroke onset at 3 stroke centers. After applying inclusion and exclusion criteria, 336 patients eligible for the TRACE‐III study were included in the final analysis (median age 65 years; interquartile range 55–73; 63.7% male) (Figure 1). Table 2 outlines the key baseline characteristics of our cohort, along with those from the TRACE‐III and TIMELESS trials.

**TABLE 2:** Demographic and clinical characteristics of the present study, TRACE‐III and TIMELESS trial.

Characteristic	Present study (*n* = 336)	TRACE‐III TNK group(*n* = 264)	TIMELESS TNK group (*n* = 228)	TIMELESS Placebo group (*n* = 230)	*p* *	*p* **	*p* ***
Median age (IQR)—year	65 (55–73)	67 (58–75)	72 (62–79)	73 (63–82)	—	—	—
Age, year							
< 80	296/336 (88.1)	236/264 (89.4)	174/226 (77.0)	156/229 (69.4)	0.618	< 0.001	< 0.001
≥ 80	40/336 (11.9)	28/264 (10.6)	52/226 (23.0)	73/229 (30.6)			
Male sex	214 (63.7)	183 (69.3)	106 (46.5)	107 (46.5)	0.065	< 0.001	< 0.001
Hypertension	198 (58.9)	177 (67.0)	—	—	0.041	—	—
Diabetes mellitus	80 (23.8)	69 (26.1)	—	—	0.513	—	—
Atrial fibrillation	61 (19.6)	49 (18.6)	—	—	0.342	—	—
mRS score before stroke^†^							
0	303 (90.2)	230 (87.1)	—	—	0.342	—	—
1	33 (9.8)	34 (12.9)	—	—			
Median NIHSS score at admission (IQR)^‡^	13 (9–17)	11 (7–15)	12 (8–17)	12 (8–18)	—	—	—
Median time from onset to treatment (hour, IQR)	12.5 (5.6–15.8)	12.3 (8.5–16.4)	12.8 (9.3–16.3)****	13.0 (8.8–17.2)****	—	—	—
Median volume of ischemic core (IQR, mL)	17.7 (6.3–29.8)	16.4 (5.7–28.4)	7.0 (0.0–22.5)	5.0 (0.0–15.0)	—	—	—
Median volume of hypoperfused lesion (IQR, mL)	114.5 (75.1–186.4)	119.1 (79.8–177.2)	92.0 (56.0–130.0)	93.0 (50.0–144.0)	—	—	—
Occlusion site							
Internal carotid artery	133 (39.6)	87 (33.0)	20 (8.8)	17 (7.4)	0.053	< 0.001	< 0.001
M1 segment	153 (45.5)	119 (45.1)	110 (48.2)	117 (50.9)			
M2 segment	50 (14.9)	58 (22.0)	89 (39.0)	84 (36.5)			
Other	—	—	9 (3.9)	12 (5.2)			
ASPECTS, median (IQR)	8.0 (7.0–9.0)	—	8.0 (6.0–8.0)	8.0 (7.0–9.0)	—	—	—

*Note:* Data are mean (SD), median (IQR), or *n* (%). IQR indicates interquartile range.

* A comparison between our cohort and the TRACE‐III TNK group.

** A comparison between our cohort and the TIMELESS TNK group.

*** A comparison between our cohort and the TIMELESS Placebo group.

**** The approximate value.

^†^ modified Rakin score.

^‡^ interquartile range.

Compared to patients in TIMELESS trial, patients in our cohort and TRACE‐III trial were younger, more male, higher proportion of patients < 80 years old, lower often M2 segment occlusion. The distribution of occlusion site was significantly different between our cohort and other 2 trials (*p* < 0.05). In our cohort, the median score on the baseline NIHSS was 13 (IQR 9–17) and the median ASPECT score was 8 (IQR 7–9). The median interval between the time that the patient was last known to be well and arterial puncture was 7.3 h (IQR 5.6–10.8). The median volume of ischemic core and median volume of hypoperfused lesion on CT perfusion imaging was 17.7 mL (IQR 6.3–29.8) and 114.5 mL (IQR 75.1–186.4), respectively.

### 3.2 Clinical Outcomes

**FIGURE 2:**
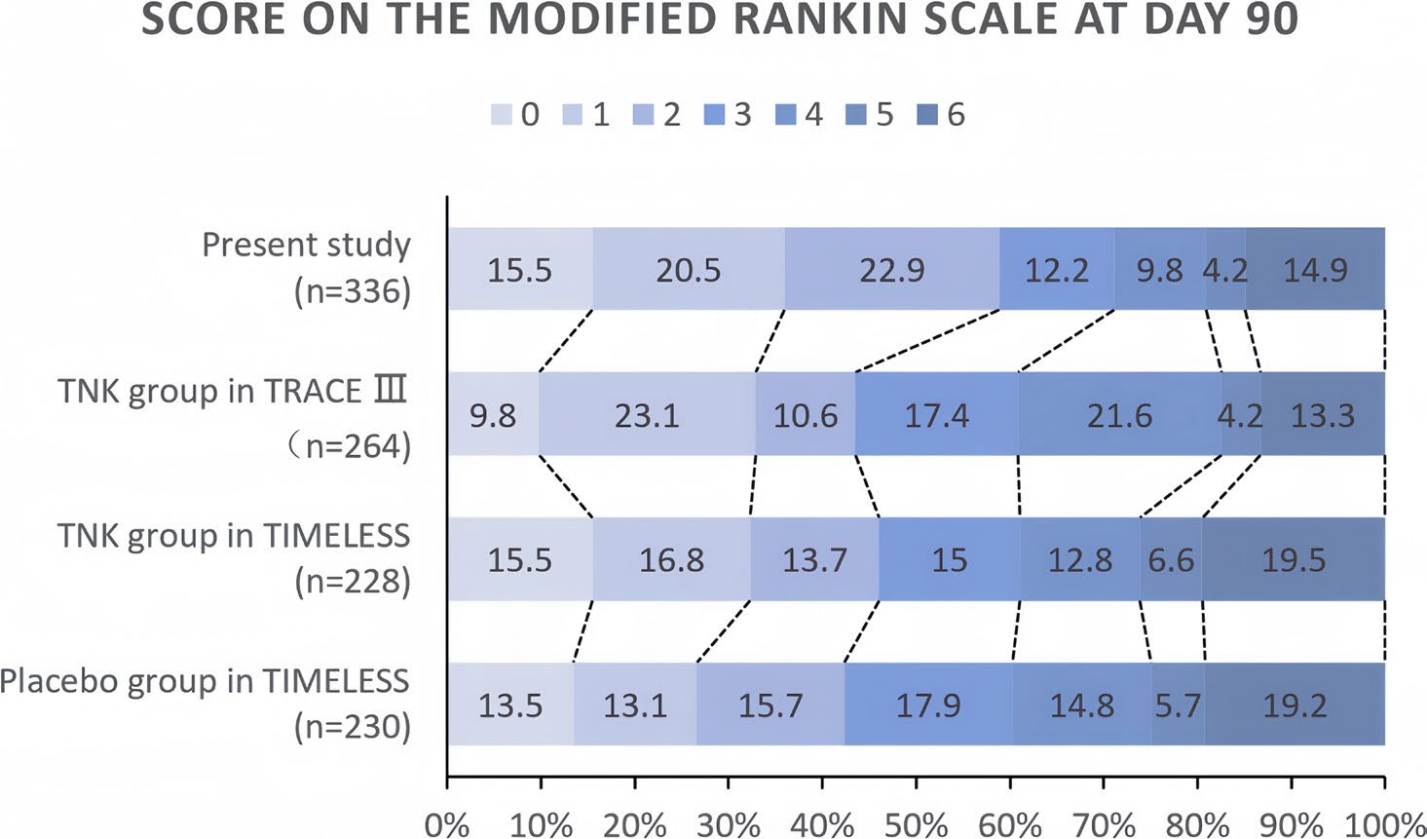
Distribution of Modified Rankin Scale scores (0–6, with higher scores indicating greater disability) at 90 days for the present study, TRACE‐III TNK group, and TIMELESS TNK and placebo groups. The numbers within the bars represent the percentage of patients with each score; percentages may not total 100 due to rounding.

In our cohort, 58.9% of patients achieved good functional outcome (mRS 0–2 at 90 days), significantly higher than the TRACE‐III TNK group (43.6%, *p* < 0.001), the TIMELESS TNK group (46.0%, *p* = 0.003) and the TIMELESS placebo group (42.4%, *p* < 0.001). However, there was no significant difference in excellent functional outcome (mRS 0–1 at 90 days) between our cohort and the TRACE‐III TNK group and the TIMELESS TNK group (36.0% vs. 33.0% vs. 32.3%; all *p* > 0.05), but higher than the TIMELESS placebo group (26.6%, *p* = 0.019, Figure 2). The ordinal score on the mRS at day 90 revealed significant difference between our cohort and TRACE‐III (*p* < 0.001) and the TIMELESS placebo group (*p* = 0.012). The median mRS score at 90 days in our cohort was 2 (IQR 1–4), significantly lower than that of the TRACE‐III TNK group (3 [IQR 1–4], *p* = 0.026), the TIMELESS TNK group (3 [IQR 1–5], *p* = 0.037) and the TIMELESS placebo group (3 [IQR 1–4], *p* = 0.003).

### 3.3 Imaging Outcomes

At the end of the procedure, the rate of successful angiographic recanalization did not significantly differ between our cohort and the two TIMELESS groups (90.2% vs. 89.1% vs. 85.4%; *p* = 0.713 and *p* = 0.105, respectively; Table 3). In our cohort, a total of 315 patients underwent MRA or CTA follow up post MT, early‐stage reperfusion was confirmed in 217 cases (86.0%), which was significantly higher than in the TRACE‐III TNK group (27.9%, *p* < 0.001), the TIMELESS TNK group (76.7%, *p* = 0.007), and the TIMELESS placebo group (63.9%, *p* < 0.001).

**TABLE 3:** Clinical and radiological outcomes of the present study, TRACE‐III, and TIMELESS trial.

Outcomes	Present study (*n* = 336)	TRACE III, tenecteplase (*n* = 264)	TIMELESS, tenecteplase (*n* = 228)	TIMELESS, placebo (*n* = 230)	*p* *	*p* **	*p* ***
mRS score of 0–2 at 90 days^†^	198/336 (58.9)	115/264 (43.6)	104/226 (46.0)	97/229 (42.4)	< 0.001	0.003	< 0.001
mRS score of 0–1 at 90 days^†^	121/336 (36.0)	87/264 (33.0)	73/226 (32.3)	61/229 (26.6)	0.435	0.364	0.019
Ordinal distribution of mRS scores at 90 days							
0	52/336 (15.5)	26/264 (9.8)	35/226 (15.5)	31/229 (13.5)	< 0.001	0.058	0.012
1	69/336 (20.5)	61/264 (23.1)	38/226 (16.8)	30/229 (13.1)			
2	77/336 (22.9)	28/264 (10.6)	31/226 (13.7)	36/229 (15.7)			
3	41/336 (12.2)	46/264 (17.4)	34/226 (15.0)	41/229 (17.9)			
4	33/336 (9.8)	57/264 (21.6)	29/226 (12.8)	34/229 (14.8)			
5	14/336 (4.2)	11/264 (4.2)	15/226 (6.6)	13/229 (5.7)			
6	50/336 (14.9)	35/264 (13.3)	44/226 (19.5)	44/229 (19.2)			
Median score on the mRS at 90 days (IQR)	2 (1–4)	3 (1–4)	3 (1–5)	3 (1–4)	0.026	0.037	0.003
Angiographic reperfusion at the end of the procedure (mTICI grade 2b to 3)	303/336 (90.2)	—	156/175 (89.1)	152/178 (85.4)	—	0.713	0.105
Early‐stage reperfusion	271/315 (86.0)	69/239 (27.9)	148/193 (76.7)	124/194 (63.9)	< 0.001	0.007	< 0.001
Symptomatic intracranial hemorrhage^‡^	18/336 (5.4)	8/264 (3.0)	7/218 (3.2)	5/214 (2.3)	0.158	0.225	0.084
Parenchymal hematoma within 72 h after treatment							
Type 1	4/336 (1.2)	—	2/218 (0.9)	1/214 (0.5)	—	0.762	0.384
Type 2	20/336 (6.0)	—	8/218 (3.7)	6/214 (2.8)	—	0.231	0.090
Death within 90 days	50/336 (14.9)	35/264 (13.3)	43/218 (19.7)	39/214 (18.2)	0.571	0.139	0.302

*Note:* Data are mean (SD), median (IQR), or *n* (%). IQR indicates interquartile range.

^†^ modified Rakin score.

^‡^ interquartile range.

* A comparison between our cohort and the TRACE‐III TNK group.

** A comparison between our cohort and the TIMELESS TNK group.

*** A comparison between our cohort and the TIMELESS placebo group.

### 3.4 Safety Outcomes

There was no significant difference in 90‐day mortality rates among our cohort, the TRACE‐III TNK group, and the TIMELESS TNK and placebo groups (14.9%, 13.3%, 19.7%, and 18.2%, respectively; all *p* > 0.05). Similarly, the incidence of symptomatic intracerebral hemorrhage (sICH) within 36 h post‐treatment showed no significant variation between our cohort and the two trials (5.4%, 3.0%, 3.2%, and 2.3%, respectively; all *p* > 0.05).

## 4 Discussion

The TRACE‐III study, conducted 4.5 to 24 h after symptom onset in AIS patients with salvageable tissue on perfusion imaging, demonstrated that TNK improved 90‐day functional outcomes compared to placebo, despite a reperfusion rate of only 27.9%. In contrast, the TIMELESS trial, which also included patients within the same time window, showed no clinical benefit of TNK over placebo. A notable difference between the trials was that 77.3% of patients in the TIMELESS trial subsequently underwent MT. Interestingly, the TRACE‐III study reported favorable functional outcomes in 43.6% of patients, comparable to the TIMELESS trial (46.0%) and the DEFUSE‐3 trial (44.6%) which involved patients treated within a shorter time window (6–16 h) [13]. Considering that the perfusion imaging selection criteria were consistent across all three trials, these findings raise a important question about whether successful vascular recanalization or the use of TNK is the primary determinant of favorable clinical outcomes. In the TIMELESS trial, approximately 46% of patients achieved a favorable outcome (mRS 0–2), with the majority undergoing MT. In contrast, the TRACE‐III trial reported favorable outcomes in 43.6% of patients treated with TNK without additional MT. These results challenge the assumption of a definitive additional benefit from MT and highlight the need to better understand the interplay between thrombolytic therapy and recanalization strategies in patients with perfusion mismatch beyond 4.5 h.

In this study, we compared clinical, radiological, and safety outcomes between our cohort and the TRACE‐III and TIMELESS trials. Rapid and effective recanalization of an occluded artery is a strong predictor of favorable functional outcomes in LVO stroke [14]. We hypothesize that the high rate of successful recanalization and early‐stage reperfusion achieved by MT significantly contributes to the higher percentage of good functional outcomes observed in our cohort (57.7%) compared to the TRACE‐III (43.6%), TIMELESS TNK group (46.0%), and placebo group (42.4%). In our study, TRACE‐III‐eligible patients, mainly treated with stent retriever thrombectomy or contact aspiration, achieved a recanalization rate of 90.4%. This is comparable to the 89.1% in the TIMELESS TNK group and 85.4% in the TIMELESS placebo group. However, since approximately 23% of patients did not undergo MT in the TIMELESS, resulting in lower actual recanalization rates of 68.4% in the TNK group and 66.1% in the placebo group. Post‐MT reperfusion follow‐up using CTA or MRA confirmed that 84.8% of cases in our cohort achieved early‐stage reperfusion, compared to 20.1% in TRACE‐III and 76.7% and 63.9% in the TIMELESS TNK and placebo groups, respectively. Successful recanalization and early‐stage reperfusion are believed to be critical for favorable outcomes, given the similarities in age, NIHSS score, baseline ASPECT score, and treatment time between our cohort and the aforementioned trials.

However, a challenge remains with the percentage of patients achieving an excellent functional outcome (mRS 0 to 1 at 90 days) in our cohort being 34.3%, similar to the TNK groups in TRACE‐III (33.0%) and TIMELESS (32.3%). These findings suggest that MT does not provide a higher rate of excellent outcomes compared to TNK. Several factors may account for this result. Our cohort had higher baseline NIHSS scores than TRACE‐III, and despite a shorter time from symptom onset to treatment, exhibited similar ischemic core and hypoperfusion lesion volumes. This indicates faster infarct growth and poorer collateral circulation, which are crucial for a worse prognosis. Additionally, the similar excellent outcome rate in TIMELESS can be attributed to smaller core infarct volumes in their patients. Furthermore, our cohort had a lower proportion of M2 occlusions and a higher proportion of ICA occlusions, indicating a larger thrombus burden, potentially leading to longer recanalization times and lower first‐pass recanalization rates, contributing to the relatively lower excellent functional outcomes.

In our cohort, the rates of post‐MT sICH and 90‐day mortality were 5.3% and 14.6%, respectively. These findings are consistent with those from the TRACE‐III and TIMELESS trials. Our study's safety profile aligns with the DAWN and DEFUSE 3 trials, where the incidence of sICH in the MT arm was 6% and 7%, and mortality rates were 19% and 14%, respectively.

## 5 Limitations

This study has several limitations that warrant discussion. First, the retrospective design introduces potential selection bias and confounding factors. Notable demographic and clinical imbalances exist between our cohort and the TRACE‐III and TIMELESS trials. Patients in our retrospective multicenter cohort and the TRACE‐III trial are generally younger, include a higher proportion of males, and have a lower likelihood of M2 segment occlusion compared to those in the TIMELESS trial. Second, patients were treated at three stroke centers, each with its own clinical protocols, leading to variations in physician decision‐making. Additionally, the lack of access to source data from the two trials hindered our ability to perform multivariable analyses or conduct propensity‐matched analyses to reduce bias. An additional limitation is the inability to conduct subgroup analyses. Finally, important issues remain unaddressed, such as the lack of core lab adjudication and potential biases in reporting clinical outcomes. These limitations may restrict the generalizability of our findings.

## 6 Conclusions

Our study demonstrates that in TRACE‐III‐eligible patients, MT can achieves higher rates of early‐stage reperfusion and tended to yield better functional outcomes compared to intravenous TNK, with comparable safety profiles. MT remains the preferred treatment for patients with LVO stroke. Nevertheless, TNK monotherapy remains a viable alternative when MT is unavailable. Further randomized controlled trial are needed to confirm these findings.

## Author Contributions

Chongyang Huang, Yanru Liu, Chengcheng Zhu, and Edgar Samaniego: analysis or interpretation of the data; drafting or revising the manuscript for intellectual content. Jiangang Zhang, Sheng Guan, and Tao Quan: analysis or interpretation of the data; drafting or revising the manuscript for intellectual content, major role in the acquisition of data. Zhen Chen, Xiaojie Fu, Sen Wei, and Kaihao Han: analysis or interpretation of the data; major role in the acquisition of data. Yueqi Zhu and Haowen Xu: analysis or interpretation of the data; drafting or revising the manuscript for intellectual content; design or conceptualization of the study; major role in the acquisition of data. All authors have read the final manuscript and approved its submission for publication.

## Ethics Statement

The study was carried out in accordance with the 1964 Declaration of Helsinki and its later amendments. It was centrally approved by the Ethics Committee of The First Affiliated Hospital of Zhengzhou University (Protocol: 2024‐KY‐406) as the leading ethics committee. Further approval was obtained from local ethics committees according to local regulations.

## Consent

The authors have nothing to report.

## Conflicts of Interest

The authors declare no conflicts of interest.

## Data Availability

The data that support the findings of this study are available from the corresponding author upon reasonable request.
